# Tripal MegaSearch: a tool for interactive and customizable query and download of big data

**DOI:** 10.1093/database/baab023

**Published:** 2021-04-26

**Authors:** Sook Jung, Chun-Huai Cheng, Katheryn Buble, Taein Lee, Jodi Humann, Jing Yu, James Crabb, Heidi Hough, Dorrie Main

**Affiliations:** Department of Horticulture, Washington State University, 45 Johnson Hall, Pullman, WA 99164, USA; Department of Horticulture, Washington State University, 45 Johnson Hall, Pullman, WA 99164, USA; Department of Horticulture, Washington State University, 45 Johnson Hall, Pullman, WA 99164, USA; Department of Horticulture, Washington State University, 45 Johnson Hall, Pullman, WA 99164, USA; Department of Horticulture, Washington State University, 45 Johnson Hall, Pullman, WA 99164, USA; Department of Horticulture, Washington State University, 45 Johnson Hall, Pullman, WA 99164, USA; Department of Horticulture, Washington State University, 45 Johnson Hall, Pullman, WA 99164, USA; Department of Horticulture, Washington State University, 45 Johnson Hall, Pullman, WA 99164, USA; Department of Horticulture, Washington State University, 45 Johnson Hall, Pullman, WA 99164, USA

## Abstract

Tripal MegaSearch is a Tripal module for querying and downloading biological data stored in Chado. This module allows site users to select data types, restrict the dataset by applying various filters and then customizing fields to view and download through a single interface. Set by site administrators, example data types include gene, germplasm, marker, map, QTL, genotype, phenotype and expression data. When querying for genes, users can restrict the gene dataset using various filters such as name, chromosome position and functional annotation. They can then customize fields to download, such as name, organism, type, chromosome position, various functional annotations such as BLAST, KEGG, InterPro and GO term. FASTA files can also be downloaded for the sequence data. Site administrators can choose from two different data sources to serve data: Tripal MegaSearch materialized views or Chado tables. If neither data source is desired, administrators may also create their own materialized views and serve them through the flexible dynamic Tripal MegaSearch query form. Tripal MegaSearch is currently implemented in several databases including the Genome Database for Rosaceae www.rosaceae.org and TreeGenes www.https://treegenesdb.org/.

## Introduction

In the era of data-driven science, opportunities to accelerate scientific discovery and translate results into knowledge that can solve issues in agriculture and medicine depend heavily on data integration. These integrated data can help uncover hidden insights and can also be used for further analyses and experiments to gain more knowledge. In addition, these integrated data need to be accessible through a web interface with a flexible query system where scientists can readily explore and download the data that they need.

Tripal (1), an ontology-based toolkit for construction of online biological databases, provides a solution for building databases that can efficiently integrate various types of biological data. It combines the GMOD Chado database schema (2) with Drupal, a popular website creation and content management software. The modular and ontology-based structure of Chado allows users to integrate new data types in the database without restructuring the schema, which is particularly advantageous in the world of fast-changing biotechnology. Drupal, with one of the largest open-source communities in the world, provides security, performance and account management and is extensible via an application programming interface (API) that allows site developers to create new PHP modules. Tripal provides a suite of Drupal modules for both back-end biological data stored in the Chado database and front-end display. In addition, the API that Tripal provides allows a site developer to create their own extension modules to share with other Tripal developers. For example, extension modules for searching data include the MainLab Chado Search (3) and Tripal ElasticSearch (4).

Most biological databases provide search pages for specific data types such as gene and QTL and provide results with more information about the gene and QTL. The majority of these search pages allow users to filter datasets by various categories, but the data field for view/download is often predefined. For example, users may only want to download primer sequences for the marker sets they refined, but the downloaded data may only provide genetic map positions and other information such as references, even though the database does have the information on the primers. In addition, when new metadata are available for the data type, such as newly associated functional ontology terms or associated markers for a gene, the search page has to be rewritten so that users can search using the new metadata and/or download the new metadata.

BioMart (5) provides a single web interface where users can perform complex queries to download data with user-defined metadata. It requires, however, data to be stored in a separate relational database schema that is compliant with BioMart definitions. Tripal databases store data in the Chado schema and would be required then to maintain two separate databases if they want to use BioMart. To address the issue, we have developed a Tripal Extension module called MegaSearch, which provides Tripal databases with advanced querying and downloading functionality without storing the data in a separate schema.

The Tripal MegaSearch module has been implemented in multiple databases including the Genome Database for Rosaceae (www.rosaceae.org, 6), CottonGen (www.cottongen.org, 7), the Citrus Genome Database (www.citrusgenomedb.org), Genome Database for Vaccinium (www.vaccinium.org), the Pulse Crop Database (www.pulsedb.org) and TreeGenes (treegene.org, 8). This MegaSearch Tripal extension module can be found along with the user documentation in the MainLab organization’s GitLab repository at https://gitlab.com/mainlabwsu and can also be accessed from https://tripal.readthedocs.io/en/latest/extensions/search.html#tripal-megasearch.

## Description

### User interface

**Figure 1.**
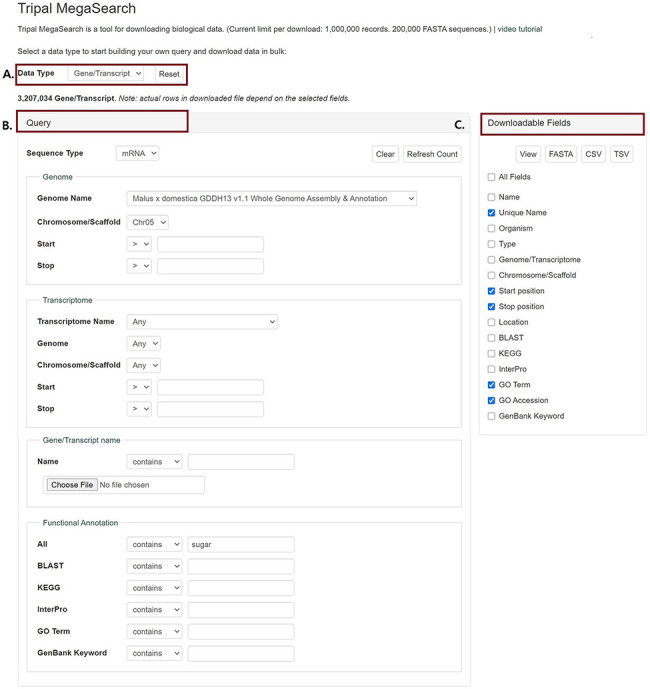
An example interface of complex static forms of Tripal MegaSearch (A). Data Type section where users can choose data type (B). Query section that provides a query form that allows users to perform complex queries using various metadata as filters (C). Downloadable Fields section where users can choose data fields to view and download.

**Figure 2.**
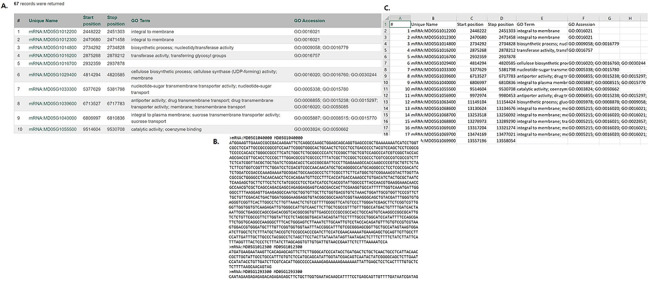
Examples of result table and downloaded files from MegaSearch (A). A result table with the chosen data fields is displayed with hyperlinks to appropriate pages such as JBrowse and gene/transcript page (B). A downloaded FASTA file for the data types with sequences, such as gene/transcript and marker (C). A CSV file that with the chosen data fields.

In MegaSearch, users first choose the data type that they are interested in, such as gene/transcript, QTL and marker (Figure 1A). The MegaSearch page has three main sections: Data type dropdown on top, Query on the left and Downloadable Fields on the right. The Query section provides a query form that allows users to perform complex queries using various metadata as filters (Figure 1B). For example, in the gene/transcript search, users can search by type, genome/transcriptome location, gene/transcript name and functional annotation terms and users can upload a file with a list of gene/transcript names as well. Once users make selections and press Refresh Count, the page returns the number of data. When users want to enter a different query, they can press Clear next to the Refresh Count. To change data type, users can simply choose a different data type in the drop down. Reset button can be used to clear the session data and reset the form. When users are done with setting up the query, they can choose downloadable fields on the right section and click either View or Download (Figure 1C). Upon clicking the View button, a table with the chosen metadata is displayed with hyperlinks to appropriate pages such as JBrowse and gene/transcript page (Figure 2A). For the data types with sequences, such as gene/transcript and marker, users can download a FASTA file (Figure 2B) with sequences as well as a CSV or TSV file (Figure 2C).

**Figure 3.**
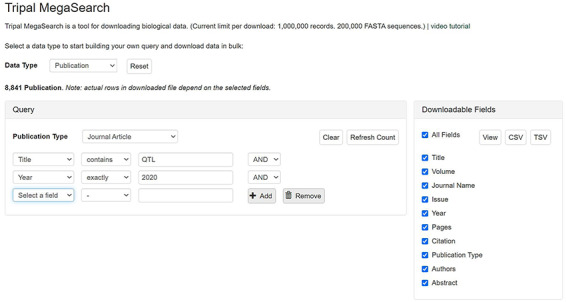
An example interface of flexible dynamic query forms of Tripal MegaSearch. The filters, pre-populated with values mapped to the underlying data source columns, can be added dynamically in this type of interface.

The query form explained above is an example of complex static forms that are available as default for gene/transcript data type. Depending on the setting, a flexible dynamic query form, on which filters can be added dynamically, can be displayed. These filters are pre-populated with values mapped to the underlying data source columns so users can filter data on each column. Users can add as many filters as they desire and combine them using ‘AND/OR’ operators. For example, in publication search, they can choose a publication type and add as many filters as they want, such as ‘Title’, ‘Citation’, ‘Year’ and ‘Authors’, to query the data they want (Figure 3).

### Technical design

Tripal MegaSearch was built on top of the MainLab Chado Search module (https://tripal.readthedocs.io/en/latest/extensions/search.html#mainlab-chado-search), which provides, programmatically, an extensive framework for creating Drupal forms and form elements. It was designed to be flexible and generic enough to be implemented on any Tripal site. To that goal, Tripal MegaSearch is able to serve data from any table in the database for either on-site display or download. To further allow users to limit returned data, a dynamic form was devised. On the dynamic form, users can add a number of filters to extract records that fulfill specified conditions. Each filter corresponds to a column of a database table so they can limit results accordingly based on the column they choose. The added conditions are then combined in the final query using logical operators, which were also exposed to the users as an option, to calculate the intersection or union from the restricted data. To limit data from the administrative perspective, an editable data definition file is employed so the site administrator can determine which table, and also which columns, to make available for the end users. Finally, if a site also adopts the MainLab Chado Loader (MCL) module (3), their data should be structured in a way that is suitable for a set of predefined materialized views to pool data. Such sites can therefore have one more option to use a preset of advanced static forms and filters to serve data.

### Software installation and customization

The Tripal MegaSearch module and user documentation can be downloaded from GitLab: https://gitlab.com/mainlabwsu/tripal_megasearch.

Software installation consists of simple steps to download and enable both the Chado Search module and the Tripal MegaSearch module, with options to populate materialized views and change settings.

The Tripal MegaSearch can access data either in materialized views or Chado base tables. A materialized view is a database object that contains data from a query. The use of materialized views allows faster retrieval of data that would otherwise require much more time to query from Chado’s highly normalized tables. Upon installation, two data definition files, one with Tripal MegaSearch materialized views and the other with Chado base tables, are available to choose from as a data source. When building a new Tripal database, one option of loading data into Chado is using the MCL module, included in the suite of MainLab Tripal Chado Data extension modules (3). The MCL provides a collection of templates for various data types and the web forms where curators can upload the data into Chado. When MCL is used to upload the data into Chado, the materialized views for Tripal MegaSearch can be employed without further customization. However, data definition files can be copied into a new file and modified to include any new tables or materialized views to make them available for query through a dynamic form. In addition, the static query forms can be modified if it is desirable to add or delete any filter. The detailed instructions are available in the README document that accompanies the module.

### Administration page

**Figure 4.**
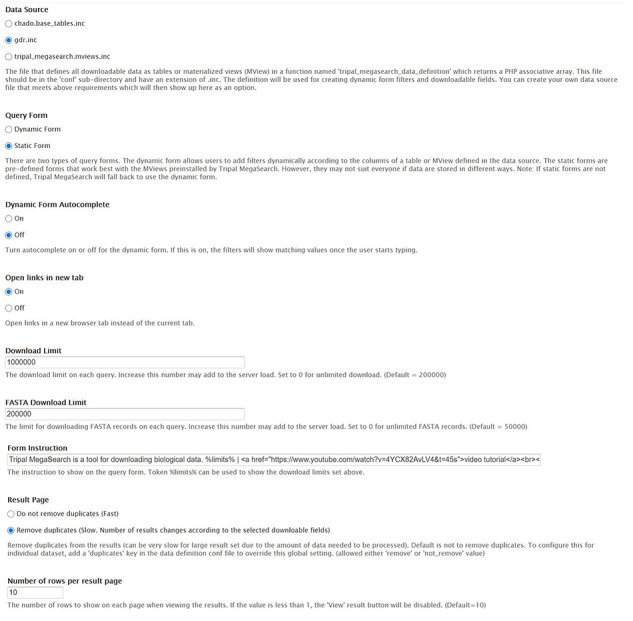
The Tripal MegaSearch Administration page that supports configuration of various settings.

The Tripal MegaSearch Administration page supports configuration of various settings (Figure 4). The Data Source section lets administrators choose a data definition file that decides which table(s) to pull data from. Administrators can then choose between two types of query forms. The dynamic form allows users to add filters incrementally as needed. The static forms are predefined forms that work best with the materialized views preinstalled by Tripal MegaSearch. The Dynamic Form Autocomplete section lets administrators choose to turn autocomplete on or off for the dynamic form. If it is on, the text filters will show matching values once a user starts typing. Open links in new tab section lets administrators to choose links from the result table to a new browser instead of the current tab. Administrators can also set limits for table and FASTA downloads, add form instructions, and set the number of rows to show when displaying the result on-site. There is also an option to remove duplicate rows, which vary depending on the display/download fields that users choose, from the results. For example, a marker has positions in multiple genetic maps; there will be multiple rows per marker in the downloaded tables even when the map position was not selected as downloadable fields. Selecting the ‘remove duplicates’ option in the administration page ensures the result table will have unique rows depending on the columns chosen.

## Conclusion and future direction

Tripal MegaSearch module provides a powerful search and retrieval functionality that can be applied to any type of data stored in the Chado schema of Tripal databases. We will continue to improve the functionality upon users’ request. One of the future functionality additions includes enabling web services so that the queried data are accessible by computers as well as humans. Another functionality is retrieving data in additional file formats such as VCF, so that users can use the data in other analysis tools without further formatting the data.
